# Autoimmunity to Collagen XVII (BP180‐Ag2) in Pemphigoid Associated With Parkinson’s Disease

**DOI:** 10.1155/crdm/3649019

**Published:** 2026-01-30

**Authors:** Ricardo Cid-Puente, Ingrid-Gabriela Ornelas-Ramírez, Lorena González-Herrera, Juan-José Bollain-y-Goytia, Esperanza Avalos-Diaz, Rafael Herrera-Esparza

**Affiliations:** ^1^ Universidad Autonoma de Zacatecas, Zacatecas, Mexico, uaz.edu.mx

**Keywords:** anti–BP180-Ag2, bullous pemphigoid, collagen XVII, epitope spreading, Parkinson’s disease

## Abstract

The association between Parkinson’s disease and autoimmune disease is rare; in our population, there is 1 case per 10,000 inhabitants. Bullous pemphigoid has a much lower incidence, and consequently, the association of Parkinson’s disease and bullous pemphigoid is rarer. We present the case of an 84‐year‐old patient with a 16‐year history of Parkinson’s disease, treated with rotigotine, levodopa, and carbidopa. The patient spontaneously developed tense blisters that spread to the trunk and extremities within 1 year of the first occurrence of dermatological symptoms. A lesional biopsy revealed a subepidermal blister with inflammatory infiltrates, and immunofluorescent evaluation of the biopsy revealed immune deposits of IgG at the basement membrane. The serum displayed antibasement membrane autoantibodies that reacted with monkey esophagus tissue, and immunofluorescence revealed that the patient was positive for antineuronal antibodies that reacted with mouse brain tissue. The molecular reactivity of the serum and fluid obtained from a bulla was positive for the BP180‐Ag2 antigen, as determined by ELISA. Additionally, six Parkinson’s serum samples without pemphigoid disease were tested as controls, and only one serum sample was reactive to BP180‐Ag2. A critical review of the possible pathogenic mechanisms of this rare association is discussed.

## 1. Introduction

Bullous pemphigoid (BP) is a chronic autoimmune disease characterized by subepidermal blistering that can occur especially in elderly individuals, and these patients experience exacerbations and remissions. Clinically, the disease presents as a pruritic dermatosis with large, tense blisters and bullae spread on the trunk and extremities. Other varieties of pemphigoid include pemphigoid of the mucosa, herpes gestationis, linear IgA disease, anti–laminin *γ*1/anti‐p200 pemphigoid, pemphigoid associated with renal failure, and drug‐induced pemphigoid [1].

This autoimmune disease is associated with the histocompatibility antigen HLA‐DQB1∗03:01 [2]. From a histological point of view, in 1953, Walter F. Lever characterized the disease as “*a subepidermal blistering disease, associated with an intense inflammatory infiltrate*”; then, in the 1960s, Jordon and Beutner demonstrated the presence of autoantibodies against dermal‒epidermal junctions via immunofluorescence, and these autoantibodies were found to be responsible for inducing separation of the dermis from the epidermis and for triggering inflammatory infiltrates [3].

The characterization of the antigens was performed in the 1980s by J. Stanley, who recognized the BP 230‐kDa protein from the hemidesmosome as one of the main antigens; in parallel, LA Diaz’s group demonstrated another autoantibody directed against a hemidesmosomal protein of 180 kDa [4–6]. The latter antigen corresponds to the 16th domain of collagen XVII, which is currently referred to as the BP180‐Ag2 antigen; this structural component is essential for maintaining the dermal‒epidermal junction. In brief, patients with BP produce specific autoantibodies against BP180‐Ag2, BP230‐Ag1, or both antigens simultaneously [6, 7].

On the other hand, Parkinson’s disease is a neurodegenerative pathology that affects the fine control of movement; its main symptoms are tremor at rest, bradykinesia with altered posture, and rigidity. This disease affects 1 in every 1000 inhabitants, and its incidence increases with age. Its etiology is complex and includes genetic, environmental, and cellular aging factors; in some cases, it is associated with immune dysfunction [8].

Currently, the cause of this pathogenic association remains unsolved. Intriguingly, some patients with Parkinson’s disease may present autoantibodies against BP180‐Ag2 or against BP230‐Ag1 without any skin disease; in contrast, most patients with BP lack neurological symptoms despite having autoantibodies against hemidesmosomal proteins [9–12]. However, in patients who have clinical expression of both pathologies, the following conceptual question arises: *What came first, the chicken or the egg*?

Because this interesting clinicopathological association is not frequent, we report a patient with Parkinson’s disease who subsequently developed symptoms of BP and autoantibodies against the basement membrane specific to the BP180‐Ag2.

## 2. Case Presentation

We report an 84‐year‐old patient with a 16‐year history of Parkinson’s disease. The neurodegenerative problem began at the age of 68 years, when he had noticed uncoordinated and progressive movements of the hands, such as a fine tremor linked to voluntary movements, which then became continuous and generalized at rest. He also presented alterations in gait and an expressionless appearance. For these reasons, he was admitted to neurology services and was prescribed antiparkinsonian therapy, including carbidopa and levodopa, and then treatment was supplemented with rotigotine; concurrently, he developed high blood pressure and was treated with prazosin. His Parkinson’s disease progressed gradually over the next fifteen years, and at 83 years of age, he presented with dermatological symptoms that progressed without treatment for a year until his first consultation with a dermatologist.

At his first dermatological consultation, the patient presented with dermatosis spread to the trunk and extremities, particularly the upper extremities. After 1 year of progression, the skin disease was characterized by large blisters of 1–7 cm, some of which were tense with serous content, while others were broken and covered with scabs; additionally, erythema and discrete itching occurred. He had lagophthalmos and prominent distal tremors, especially in the hands (Figures 1(a) and 1(b)).

Figure 1(a and b) Bullae and vesicles present on erythematous skin of the legs.

**Figure:**
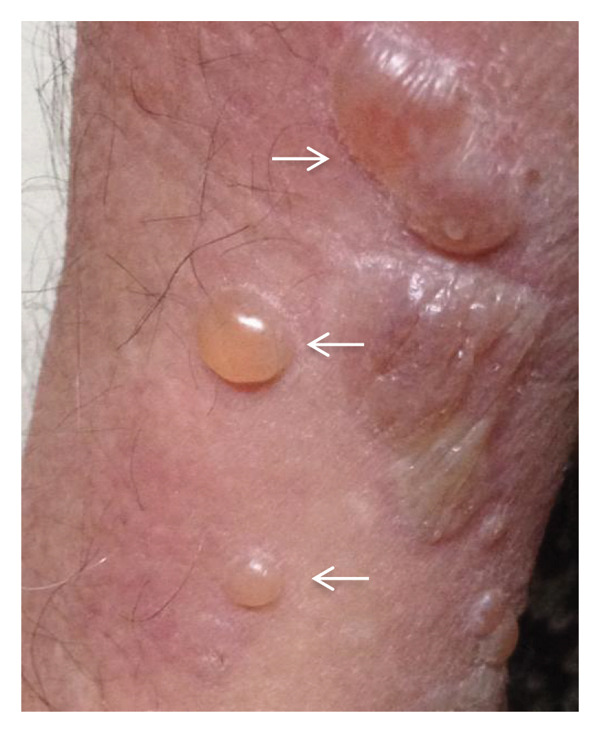
(a)

**Figure:**
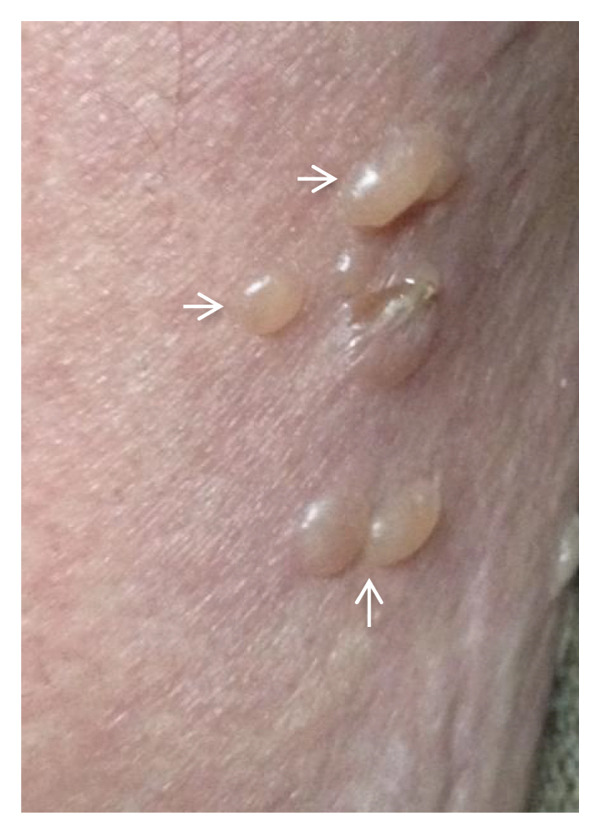
(b)

After the patient had signed an informed consent form, two skin biopsies were taken. One biopsy was performed by excision of a blister, of which one fragment was subjected to H&E staining and used for light microscopy and another fragment was utilized for immunofluorescence. The second biopsy for analysis was taken from an area of normal skin not exposed to light.

Serum samples and bulla fluid were also collected via syringe aspiration to assess the molecular reactivity against the basement membrane via indirect immunofluorescence in the monkey esophagus tissue (Euroimmun, Medizinische Labordiagnostika AG, Lübeck, Germany), and antineuronal antibodies were detected in the brain of a mouse as an antigenic source; additionally, autoantibodies against BP180‐Ag2 and BP230‐Ag1 were assessed via commercial ELISA and immunofluorescence in the BALB/c mouse brain and via western blotting.

The treatment used in this case was prednisone 30 mg/day; with this therapy, notable improvement in the following 2 months was progressively observed until the skin lesions were cleared. Clinical and serological follow‐up was performed during the following 2 years, and the lesions disappeared after 1 year of treatment. The last control consultation was held 18 months later; the skin of the patient was clean without lesions and without additional treatment (Figure 2).

Figure 2(a) Representative bullous lesion (arrows) on the right hand before treatment. (b) Note the disappearance of the lesion 2 months after starting steroid treatment.

**Figure:**
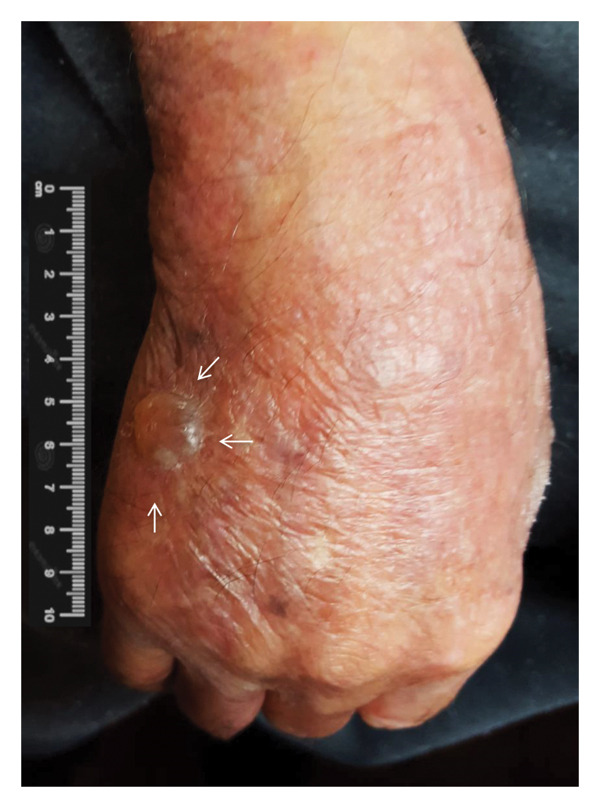
(a)

**Figure:**
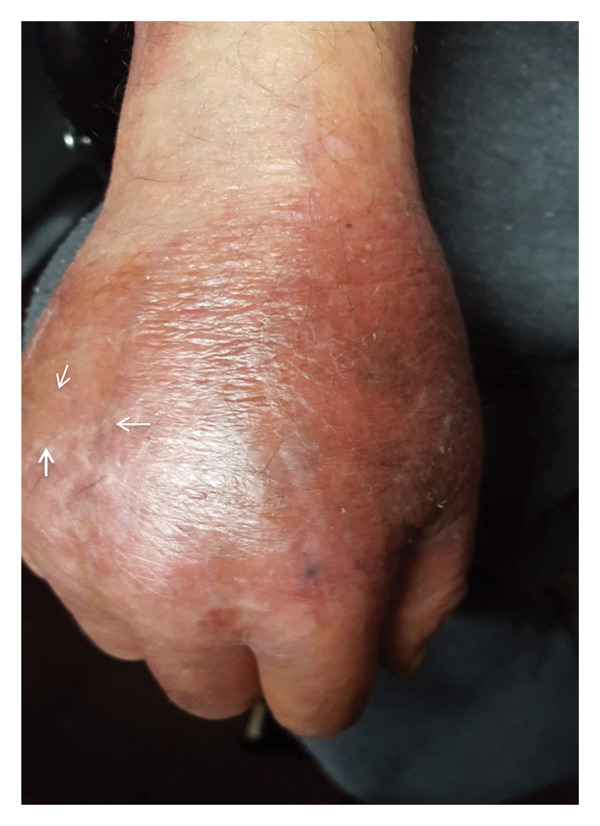
(b)

### 2.1. Skin Biopsy

H&E staining and subsequent light microscopy analysis revealed subepidermal bullae and the presence of inflammatory infiltrates composed of lymphocytic and eosinophilic infiltrates. Direct immunofluorescence of lesional skin revealed a subepidermal blister with immune deposits of IgG and C3 at the basement membrane level (Figures 3(a) and 3(b)). The uninvolved skin biopsy did not reveal blisters, inflammatory infiltrates, or IgG or C3 deposition.

Figure 3(a) H&E‐stained skin biopsy from a lesion showing a subepidermal split (arrows) with inflammatory infiltrates of mononuclear and eosinophil cells. (b) Direct immunofluorescence of perilesional and uninvolved skin from the patient revealed linear IgG and C3 deposition on the basement membrane (arrows).

**Figure:**
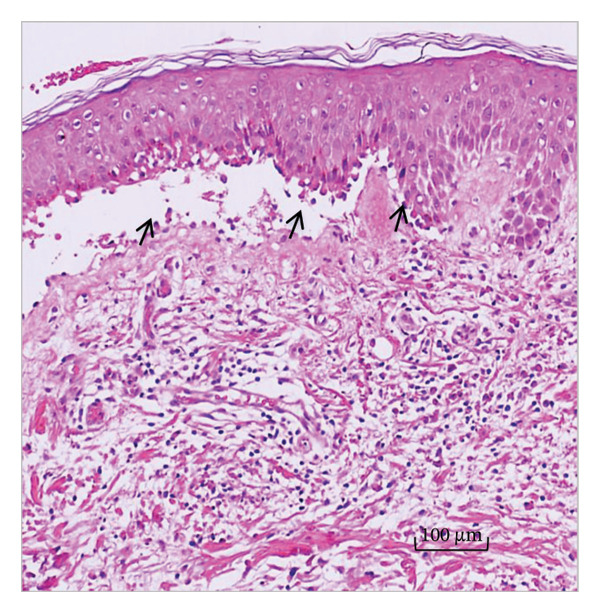
(a)

**Figure:**
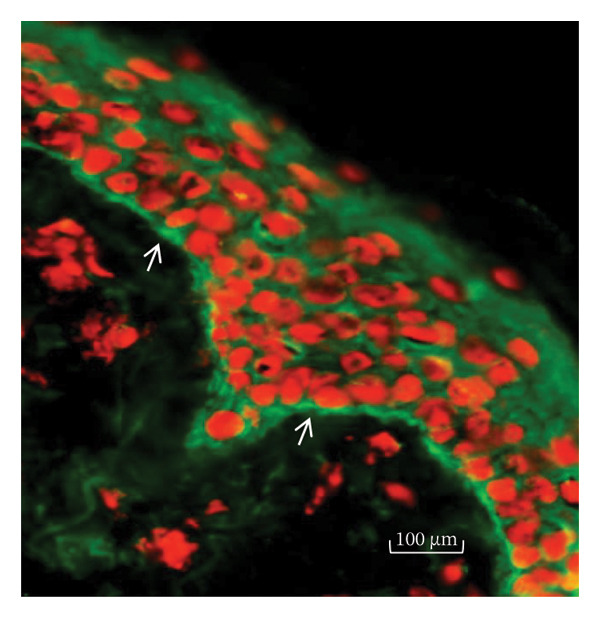
(b)

### 2.2. Serology

BP IgG autoantibodies were present in the basement membrane of the monkey esophagus used as an antigenic source; similarly, the blister fluid was also positive for the basement membrane autoantibodies, and the patient’s serum and bulla fluid tested positive for BP180‐Ag2 and negative for BP230‐Ag1, as determined by ELISA. Additionally, the patient’s serum recognized the collagen XVII filaments from the neuronal bodies of the mouse brain. Western blot analysis using human skin commercial extract (Santa Cruz sc‐363777) and BALB/c brain extract (Santa Cruz sc‐2253) revealed that a 180‐kDa protein was recognized by the serum (Figures 4(a), 4(b), and 4(c)).

Figure 4(a) Bullous pemphigoid autoantibody (IgG) detected by indirect immunofluorescence reacted against the basement membrane of monkey esophagus tissue. (b) Antineuronal antibodies detected by indirect immunofluorescence in the mouse brain tissue. (c) Western blot of human skin and BALB/c brain extracts. The molecular weight standards (Bio‐Rad) are shown in the leftmost lane. Lane 1 contains human skin extract, and Lane 2 contains BALB/c brain extract. A band of ≈180 kDa was recognized by the BP serum (<).

**Figure:**
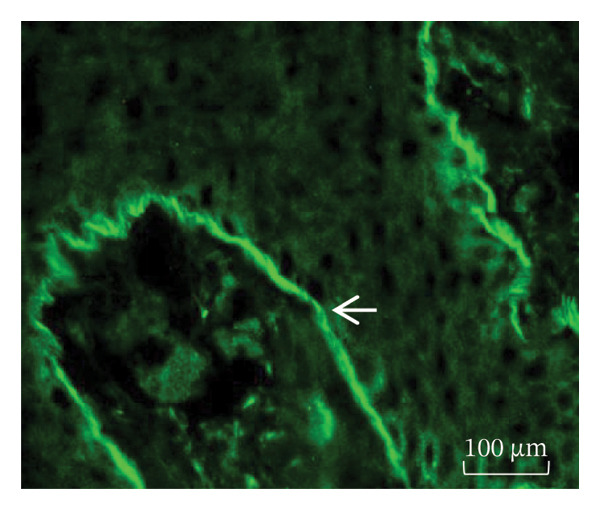
(a)

**Figure:**
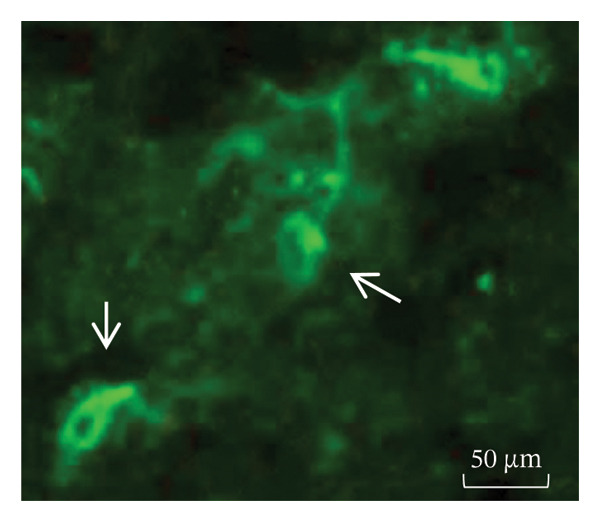
(b)

**Figure:**
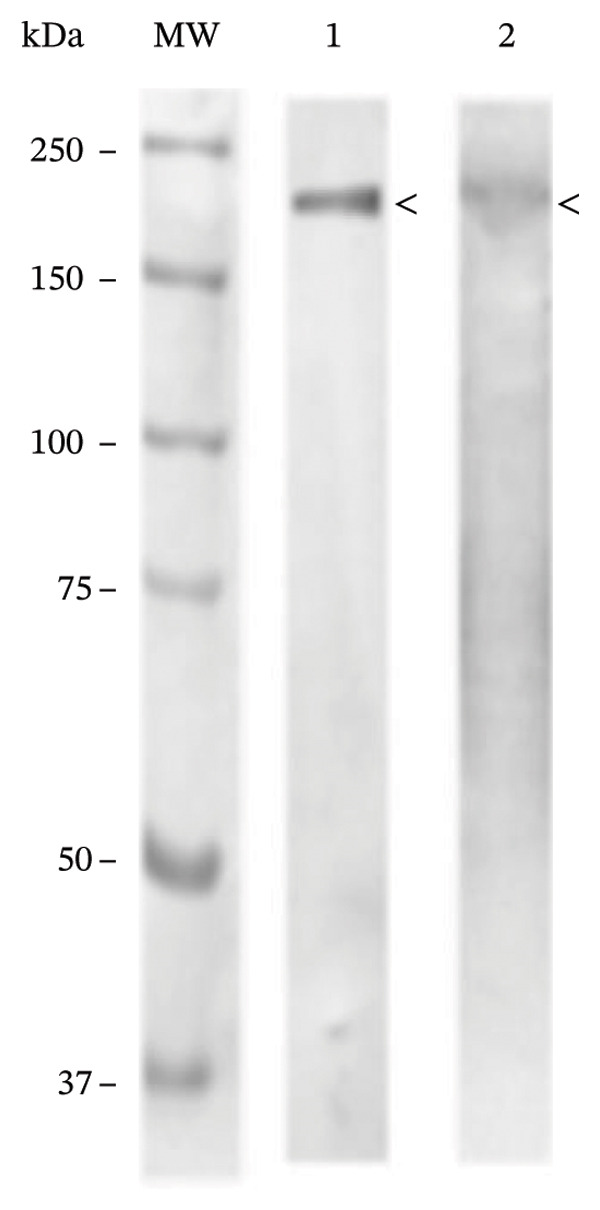
(c)

### 2.3. Control Serum

Samples from 6 patients with Parkinson’s disease without skin involvement were obtained from the blood bank and used as controls, and these patients had previously signed a routine consent form. According to the ELISA results, only one serum sample was reactive for BP180‐Ag2, and the remaining control serum samples were negative.

## 3. Discussion

The association between Parkinson’s disease and autoimmune disease is rare, although neurological disorders affect 1% of individuals over 65 years of age globally. The incidence rate is 1 per 10,000 inhabitants; however, small variations in the data may occur depending on the ethnic group and the methodology used for data collection [9, 10]. BP has a much lower incidence, and according to a meta‐analysis, it affects 0.0082/10,000 inhabitants [11]; consequently, the co‐occurrence of both pathologies is less common [12].

The BP180‐Ag2 antigen is a transmembrane protein that is a structural component of the dermoepidermal junction and corresponds to a collagen XVII domain, and patients with BP produce autoantibodies against BP180‐Ag2 [13]. In mammals, an isoform of this protein is also present in neurons of the central nervous system, particularly in the human brain, which is expressed in the pyramidal neurons of the motor nucleus of Betz and substantia nigra, cortex, hippocampus, amygdala, cerebellum, retina, and olfactory bulb. At the cellular level, it is frequently clustered with lipofuscins of the neuronal body and is also present in the proximal axons of neurons from different regions of the brain; consequently, there is a structural and molecular basis that may explain the relationship between the shared autoimmunity between BP and Parkinson’s disease [14–17].

Additionally, the molecules encoded by the HLA‐DQB1∗03:01 allele participate in binding the epitopes from the ectodomain of the BP180 antigen (collagen XVII), which is shared by the neurons and skin; therefore, both common isoforms may play important roles in BP and in some neurodegenerative diseases with associated autoimmunity, such as Parkinson’s disease, which can be explained by the phenomenon of “epitope spreading.” This mechanism involves the diversification of epitope specificity from the primary dominant epitope of the same protein (intramolecular) or other proteins (intermolecular) [18, 19].

Autoantibodies against collagen XVII in BP are pathogenic to the skin, as they induce blisters in patients as well as in experimental models [20, 21]; however, their pathogenicity in Parkinson’s disease is not clear because neuroinflammation causes degeneration of neuronal areas that affect the fine control of movement, which in turn releases different proteins that likely leak from the blood‒brain barrier and may trigger autoantibody production. Therefore, the main question is as follows: Are the anti‐BP autoantibodies truly neuropathogenic, or are they biomarkers of neurodegeneration? Alternatively, these autoantibodies may be irrelevant and could represent a simple epiphenomenon [22].

With respect to the drugs used in Parkinson’s disease therapy as potential pemphigoid triggers, we must note that the depletion of dopaminergic neurons is the disease hallmark, and some antidiabetic drugs, such as vildagliptin, are used in Parkinson’s disease because their dopaminergic effects apparently induce neuroprotection [23]. However, one side effect of this family of dipeptidyl peptidase‐4 inhibitors, which includes vildagliptin, is associated with drug‐induced BP [24]. In our case, this possibility was ruled out since our patient was not treated with gliptins [25].

In our patient, the application of rotigotine patches was temporary, and as a result, the bullous lesions persisted until he was treated with steroids [26]. The main side effect of rotigotine, a dopamine agonist, is a contact dermatitis reaction at the site of application of the transdermal patch at the cutaneous level; therefore, skin irritation usually disappears once the patch is removed.

The patient in this case study took levodopa and carbidopa. A similar case of a patient with Parkinson’s disease taking these drugs has been reported, and that patient developed bullous eruption as a rare autoimmune condition; however, the reported case of BP was attributed to a dipeptidyl peptidase inhibitor that he received for Type 2 diabetes, which was not the case of our patient [27]. Consequently, the antiparkinsonian drug used by our patient was not the cause of the blistering. In the case of prazosin, which the patient received as a treatment for high blood pressure, there were no reports of such pathogenic associations.

A case of BP presented in a patient with preexisting Parkinson’s disease. The patient’s serum contained anti–BP180‐Ag2 antibodies, which correspond to a domain of collagen XVII. This serum recognized a similar protein in the human skin and the mouse brain extract, and the cause of this shared reactivity is likely linked to intramolecular epitope spreading between the skin and neuronal isoforms of collagen XVII. However, it is difficult to ascertain whether this phenomenon in Parkinson’s disease is pathogenic or just an epiphenomenon. Nevertheless, in patients with Parkinson’s disease or another neurodegenerative disease, the periodic monitoring of BP180/BP230 autoantibodies is recommended.

## Funding

The authors received no specific funding for this work.

## Ethics Statement

This study was conducted in accordance with the guidelines of the Declaration of Helsinki (1964) and was approved by the State Bioethics Council of the State of Zacatecas, Mexico, under number CNBMX‐CEB‐32‐20111005/001/25.

## Consent

The patient’s relatives were informed of the study and procedures and signed an informed consent form and the authorization for publication of the clinical photographs.

## Conflicts of Interest

The authors declare no conflicts of interest.

## Data Availability

Data are available upon request.
